# High-Quality Foaming and Weight Reduction in Microcellular-Injection-Molded Polycarbonate Using Supercritical Fluid Carbon Dioxide under Gas Counter Pressure

**DOI:** 10.3390/polym16182674

**Published:** 2024-09-23

**Authors:** Yogi Hendra Agustion, Shia-Chung Chen, Ching-Te Feng, Bermawi Priyatna Iskandar

**Affiliations:** 1 Master Program in Industrial Engineering and Management, Faculty of Industrial Technology, Bandung Institute of Technology, Bandung 40132, Indonesia; yogi.agustion@gmail.com; 2 Department of Mechanical Engineering, College of Engineering, Chung Yuan Christian University, Taoyuan 32023, Taiwan; g10873063@cycu.edu.tw; 3 R&D Center for Smart Manufacturing, Chung Yuan Christian University, Taoyuan 32023, Taiwan; 4 Industrial Engineering Department, Faculty of Industrial Technology, Bandung Institute of Technology, Bandung 40132, Indonesia; bermawi@itb.ac.id

**Keywords:** microcellular injection molding, carbon dioxide, gas counter pressure, foaming cell size, foaming cell density, weight reduction

## Abstract

Microcellular injection molding (MuCell^®^) using supercritical fluid (SCF) as a foaming agent to achieve weight reduction has become popular in carbon emission reduction. In the typical MuCell® process, SCF N_2_ is commonly used. Although SCF CO_2_ exhibits high solubility and can achieve a high weight reduction, controlling the foaming is not easy, and its foaming cells are usually larger and less uniform, which limits its industrial application. Our previous studies have shown that gas counter pressure (GCP) can improve the foaming quality effectively. Here, we investigated whether or not the CO_2_ SCF foaming quality could be improved, and weight reduction was achieved for polycarbonate (PC) material. This is quite important for the electronics industry, in which most of the housing for devices is made of PC materials. MuCell^®^ was subjected to molding experiments using the parameters of the SCF dosage, melt temperature, mold temperature, and injection speed. The results revealed that using CO_2_ gas for the PC material can reduce the size of microcellular cells to 40 µm and increase the cell densities to 3.97 × 10^6^ cells/cm^3^. Using GCP significantly improved the microcellular injection-molded parts by reducing the cell size to 20.9 µm (a 45.41% improvement) and increasing the cell density to 8.04 × 10^6^ cells/cm^3^ (a 102.48% improvement). However, implementing GCP may slightly decrease the target weight reduction. This study reveals that microcellular injection molding of PC parts using SCF CO_2_ can achieve high-quality foaming and reduce the weight by about 30%.

## 1. Introduction

Injection molding is one of the most common methods employed in the plastic processing industry. Injection molding technology is evolving through combining conventional injection molding and microcellular foaming. Microcellular techniques were first introduced at the Massachusetts Institute of Technology in the early 1980s to reduce the material consumption and the weight of parts [1]. In 2001, Trexel Inc. trademarked MuCell^®^ as a microcellular injection molding process using supercritical fluid (SCF) as the foaming agent and introduced the process for commercial use [2]. When using the MuCell^®^ technique, a supercritical gas (often carbon dioxide (CO_2_) or nitrogen (N_2_)) is injected into the molten polymer and expands, filling the tool cavity and forming a cellular structure in the part [3,4]. Compared with conventional injection molding, MuCell^®^ reduces the product weight and cycle time, minimizes shrinkage and warpage, and eliminates residual stresses [5]. Owing to its advantages, MuCell^®^ contributes to low fuel consumption in vehicles and reduces their carbon footprint and environmental burden [6,7].

However, MuCell^®^ faces technological challenges that prevent its widespread use. The combined impacts of partial foaming and fountain flow during the melt-filling process cause poor foaming quality (with uncontrollable, non-uniform foaming sizes) and a poor surface appearance, with swirl marks or silver streaks [2,8]. Numerous techniques have been developed to reduce such marks, including the co-injection molding process and the gas counter pressure (GCP) system [9,10]. The GCP system is used to prevent cell foaming in the flow front during the filling stage. In the early injection stage, high-pressure gas is injected into a sealed mold cavity to establish counter pressure. During filling, the counter pressure successfully stops the single-phase polymer/gas solution from foaming. Toward the end of the filling stage, the counter gas pressure is rapidly released, causing bubbles to form in the melting of the polymer. Swirl marks and silver streaks are removed since the skin layer of the part is solidified [11]. This concept was shown schematically in detail in Figure 1 of Ref. [11]. The GCP system proved useful for improving the foaming quality of microcellular injection-molded parts in our earlier studies. Based on our previous study using thermoplastic polyurethane (TPU) material and N_2_ as the blowing agent, the best homogeneity of the cell size distribution, the smallest cell sizes, and the highest cell densities were obtained when GCP and dynamic mold temperature control were applied together [2]. A schematic of GCP restricting the foaming is available in Figure 1 of Ref. [12].

Apart from N_2_, CO_2_ is an alternative gas that can function as a blowing agent in the MuCell^®^ process and has been more extensively studied. N_2_ and CO_2_ exhibit different solubilities and foaming behaviors. N_2_, with its low solubility, produces a higher number of small cells, whereas CO_2_, with its high solubility, produces fewer and larger cells [13]. However, large cells are correlated with an increased weight reduction percentage in the final product [13,14]. As a blowing agent, CO_2_ enhances the quality of parts in terms of their lightweight construction, dimensional stability, and surface quality [15]. CO_2_ seems to have higher application potential than other physical blowing agents, such as N_2_, because it creates more refined cellular architectures in specific polymers, and it is environmentally friendly and nonhazardous [16,17]. Thus, this study used CO_2_ as the blowing agent to investigate its impact on the cell size and density in the MuCell^®^ process for polycarbonate (PC) materials. PC was selected because it is an important engineering plastic widely used and applied in electronic device housing. Foaming characteristics such as the cell size and cell size distribution, as well as cell density, are closely related to mechanical performance. A comprehensive literature analysis by Ding et al., who thoroughly examined the microcellular injection molding process, including its main features, cell formation and expansion, and recent developments. However, the impact of the processing parameters on the morphology of PC material has not been investigated, specifically in terms of cell size and density [18,19]. For quite a long time, uncontrollable foaming during the MuCell^®^ process has hindered its application. Studies have shown that GCP can improve the surface quality and produce higher cell densities, smaller cells, and good uniformity in the cell size distribution compared with conventional MuCell^®^ processes [20,21]. Thus, the present study aims to obtain high-quality foaming and achieve a reduction in the weight of the parts simultaneously for PC parts using CO_2_ as the blowing agent and employing GCP technology. Table 1 outlines studies related to the development of MuCell^®^ using SCF CO_2_ or PC materials, focusing on the foaming quality and/or weight reduction.

## 2. Materials and Methods

### 2.1. Foaming Materials and Mold Design

**Figure 1:**
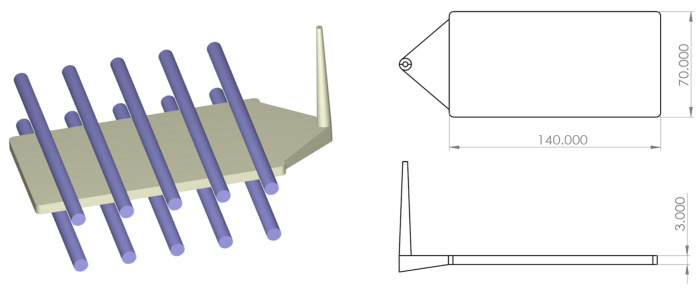
Plate-part mold used for molding experiments.

PC material (PC-110, Chi Mei Corporation) was used after drying it at 120 °C for 4 h. A thin rectangular bar was produced (140 × 70 × 3 mm in length, width, and height, respectively). This part had a fan gate with five straight symmetrical cooling channels on the core and cavity sides (Figure 1).

### 2.2. The MuCell^®^ Machine and Gas Counter Pressure System

The experiments were conducted using the ENGEL e-victory 120 (screw diameter = 40 mm; maximum clamping force = 1200 kN). This machine supports microcellular injection molding and can be used for detailed and precise experiments. An SCF generator (Trexel T-100 series provided by Trexel Inc. Hong Kong) and the GPC FX-1 (provided by Polypro Co., Ltd. Taiwan) were used. The GPC FX-1 is a cost-effective and multifunctional gas-assisted control system designed for research and industrial applications. A schematic of it is available in Figure 3 of Ref. [10].

### 2.3. The Electronic Balancer

The HUAZHI HZY-B4000 Advanced High-Precision Balance, known for its precision (maximum capacity = 4000 g; readability = 0.01 g), was used to accurately measure the weight of each produced part.

### 2.4. The Scanning Electron Microscope

**Figure 2:**
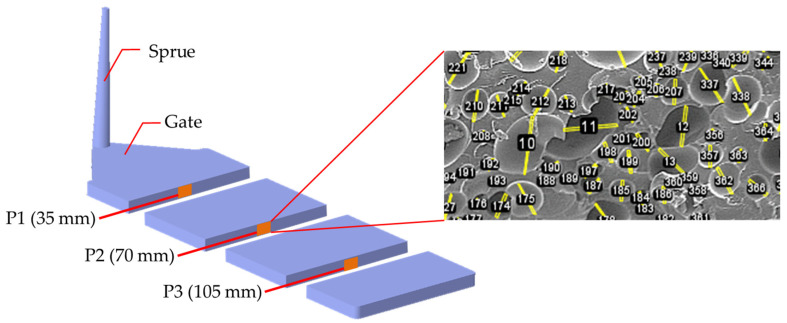
Typical SEM images of foaming bubbles at P1, P2, and P3. P1 is located 35 mm away from gate, P2 is 70 mm away, and P3 is 105 mm away, representing a location near the gate, a middle location, and a flow end location, respectively. The typical foaming morphology at P2 is illustrated.

Scanning electron microscopy (Hitachi, S-3000N, magnification range of 15×–300,000×) was performed. The test specimen surface was covered with a layer of gold foil using an ion sputter (Hitachi E-1010). SEM images of the cell structure were obtained at three positions (3.5, 7.0, and 10.5 cm from the gate) on the core side (Figure 2).

### 2.5. Experimental Parameters

The experiments were conducted in two stages, each with different processing parameters. The first stage involved the conventional MuCell^®^ experiment with processing parameter settings including SCF dosage, melt temperature, mold temperature, and injection speed measured. In this stage, the target weight reduction was fixed at 35%. The experimental design was built using Taguchi L9 orthogonal arrays (Table 2). The second stage of the experiment determined how using GCP affected the size, density, and uniformity of cells in the microcellular injection-molded part. The combination of processing parameters that yielded the optimum results during the first stage was used. If the molded parts experienced short shots and a 35% weight reduction was not achieved in the second stage, adjustments to the actual weight reduction were made to ensure that GCP could be applied throughout the entire part. Three levels of GCP and two holding times were used during the second stage.

### 2.6. Cell Size and Density Measurements

The molded parts were analyzed at three locations: P1 (front position close to the gate), P2 (position in the middle of the part), and P3 (back position far from the gate). Using the SEM image of the cell structure, the cell diameter (also known as the cell size) was measured, assuming that the cell was perfectly round. Cell diameter measurements were made on the core part of each image using ImageJ 1.54f software. To determine the cell density of a microcellular injection-molded part, Kumar and Suh [29] formulated the following equation:

$$N_{o}={\left({\frac{nM}{A}}^{2}\right)}^{\frac{3}{2}}\times \frac{1}{1-\left(\frac{\pi }{6}D^{3}\times {\left({\frac{nM}{A}}^{2}\right)}^{\frac{3}{2}}\right)},$$

where *n* is the number of cells observed in the micrograph, *M* is the magnification factor of the micrograph, *D* is the average cell diameter, and *A* is the area of the micrograph.

### 2.7. Gray Relational Analysis

Gray relational analysis (GRA) optimizes multiple responses into a single gray relational grade (GRG). GRA is used to obtain a single trend and the optimal combination of processing parameters that can simultaneously result in small cell sizes and high cell densities. The GRG is calculated as follows [30].

1. Data normalization:

For “larger is better”,

$$Z_{ij}=\frac{y_{ij}-min\left(y_{ij}\right)}{max\left(y_{ij}\right)-min\left(y_{ij}\right)},$$

For “smaller is better”,

$$Z_{ij}=\frac{max\left(y_{ij}\right)-y_{ij}}{\max\left(y_{ij}\right)-min\left(y_{ij}\right)},$$

2. The gray relational coefficient:

$$\gamma_{ij}=\frac{\Delta min+\xi \Delta max}{\Delta_{ij}+\xi \Delta max},$$

3. Calculate the GRG:

$$\bar{\gamma_{j}}=\frac{1}{m}\overset{m}{\underset{i=1}{\sum }}\gamma_{ij},$$

where *y_ij_* is the observed response value of the *i*-th response in the *j*-th experiment. Δ*_ij_* = ‖*Z_oj_* − *Z_ij_*‖ is the absolute value of the difference between the reference sequence *Z_oj_* and the actual sequence *Z_ij_*. Δ*min* = *min_i,j_* Δ*_ij_* is the minimum value of Δ*_ij_*, and Δ*max* = *max_i,j_* Δ*_ij_* is the maximum value of Δ*_ij_*. *ξ* is the distinguishing coefficient, defined within the range of 0 ≤ *ξ* ≤ 1 (the value may be adjusted based on the practical needs of the system). The commonly used value is 0.5. *m* is the number of responses.

## 3. Results and Discussion

### 3.1. First Stage of the Experiment (without GCP)

**Figure 3:**
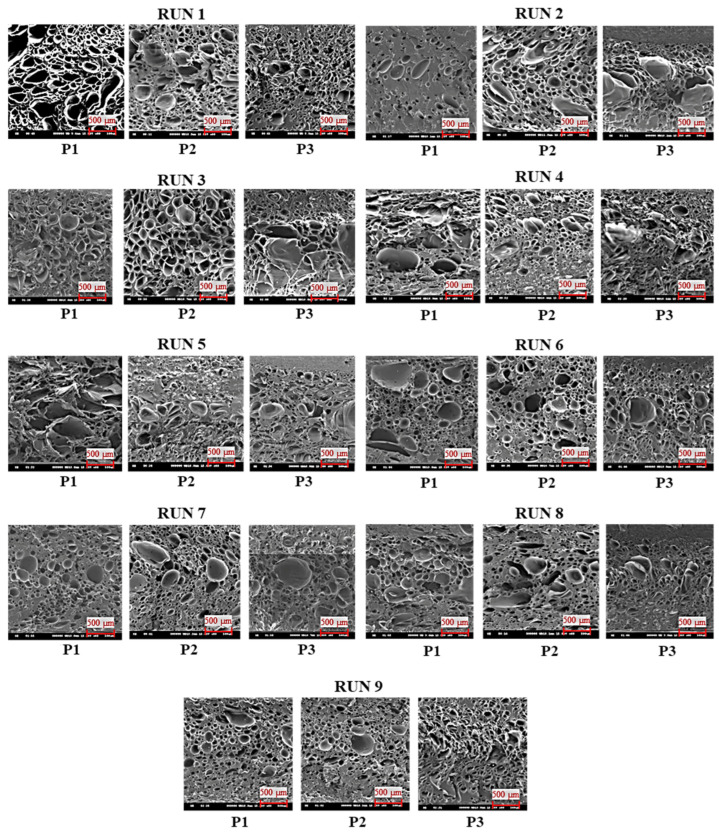
SEM images of cell structure in first stage of experiment.

The SEM images (Figure 3) of the molded parts from each run in the first stage of the experiment were analyzed using ImageJ 1.54f software to measure the cell sizes.

**Figure 4:**
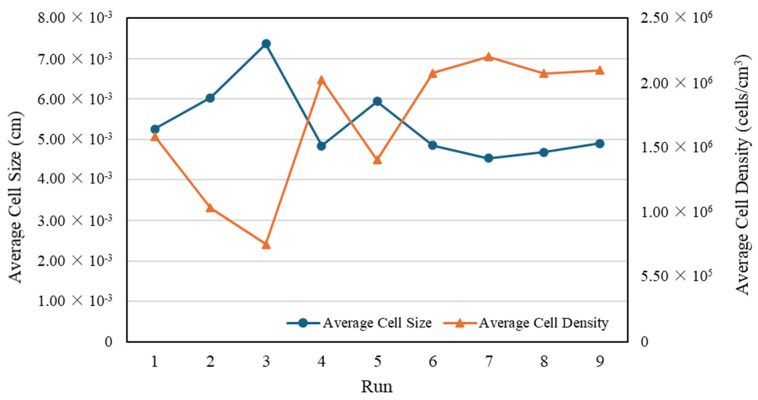
Average cell size and density in first stage of experiment.

Table 3 summarizes the cell sizes and densities for each measurement position, and Figure 4 illustrates the average cell size and density in each experimental run.

**Figure 5:**
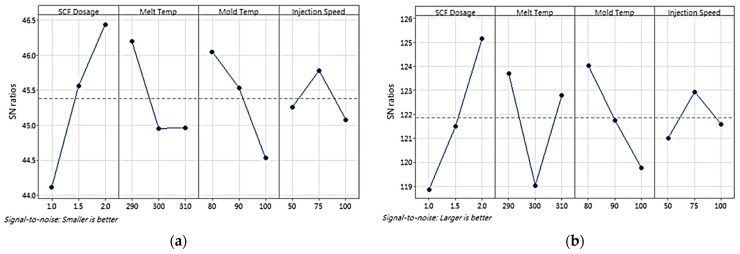
Main-effects plot of S/N ratios of (**a**) cell size and (**b**) density (P1, P2, and P3) in first stage of experiment. The dashed line represents the overall mean S/N ratio across all factor levels.

Based on the signal-to-noise (S/N) ratios of the cell size and density, a Taguchi design analysis was conducted using the Minitab version 21 software, producing a main effects plot to determine the influence of the processing parameters on the cell size and density. Furthermore, the optimum processing parameters to achieve small cell sizes and high cell densities were determined. Figure 5 shows the main effects plots of the S/N ratios for cell size and density, respectively. An analysis of variance (ANOVA) was conducted to assess the significance of each factor tested for the cell size and density. Table 4 and Table 5 present the ANOVA results for the average cell size and density, respectively.

**Figure 6:**
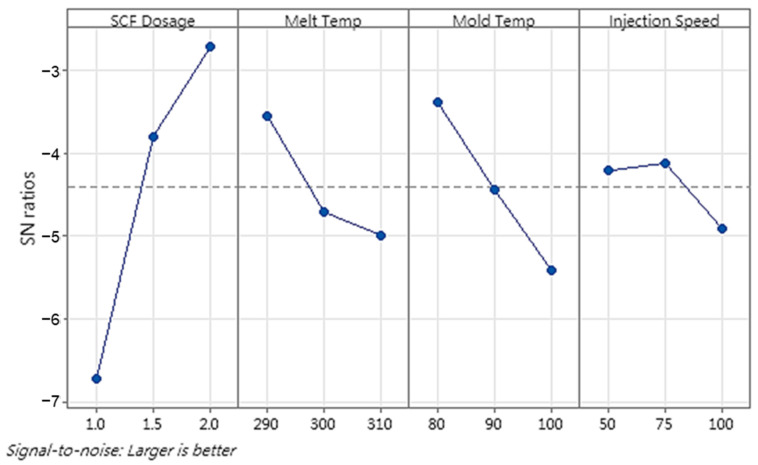
Main-effects plot of S/N ratios of the GRGs in first stage of experiment. The dashed line represents the overall mean S/N ratio across all factor levels.

The Minitab version 21 software only analyzes the cell size and density as individual responses because in the cell size analysis, the “smaller is better” response is used. However, in the cell density analysis, the “larger is better” response is used. Based on the data processing results, the parts with small cells did not necessarily have high cell densities (as shown in runs 8–9, Figure 4), and differing trends were observed in the influence of the processing parameters on the cell size and density with respect to melt temperature and injection speed. Thus, to determine the influence and optimum combination of the processing parameters for both responses, GRA was employed with six responses, namely the cell size and density at P1, P2, and P3. Table 6 lists the GRG calculation results. The GRA results showed that run 8 was the best for all responses, with the highest GRG rank. To confirm that the selected run was optimal, a Taguchi design analysis was conducted again for the GRG values using Minitab version 21. Figure 6 shows the main-effects plot of the S/N ratios for the GRG values, which indicates that the optimal combination of processing parameters was an SCF dosage of 2%, a melt temperature of 290 °C, a mold temperature of 80 °C, and an injection speed of 75 mm/s.

**Figure 7:**
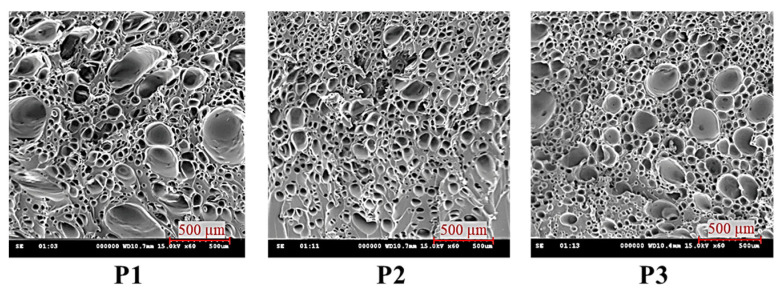
SEM images of cell structure from the validation test.

Validation tests were required because the L9 orthogonal array run order did not include the optimal combination of the processing parameters from the GRA results. This was to ensure that the combination could actually produce a smaller cell size response with a larger cell density. Furthermore, the validation test results were used as a reference to analyze the impact of the GCP on the cell size and density in the second stage of the experiment. Figure 7 shows SEM images from the validation test, and Table 7 shows a comparison between the cell size and density from run 8 as the best in the GRA and validation test. The validation test results showed improvements of 18.23% and 91.59% in the average cell size and density, respectively.

The results of the analysis revealed that a combination of processing parameters of an SCF dosage of 2%, a melt temperature of 290 °C, a mold temperature of 80 °C, and an injection speed of 75 mm/s could achieve small cell sizes and high cell densities. The results of the analysis revealed that the SCF dosage was the most significant factor in achieving small cells and high cell densities, followed by mold temperature, melt temperature, and injection speed. High SCF levels improved the nucleation process, leading to numerous small cells that were more evenly distributed. Low melt temperatures effectively limited polymer chain mobility, thereby restricting cell growth and promoting fine cell structures. Similarly, low mold temperatures contributed to the achievement of high cell densities and small cells by accelerating the cooling process, which limited the cell growth time and improved the cell density. The impact of injection speed on the cell size and density optimization was less pronounced. However, the flow dynamics in the cavity during injection molding and the 3 mm part thickness may explain this observation.

### 3.2. Second Stage of the Experiment (with GCP)

This stage was conducted to examine the impact of the pressure and holding time from the GCP on the cell size and density using the experimental design outlined in Table 8.

To ensure that GCP could be implemented on all the parts, the weight reduction limit was adjusted in each experimental run. The weight reduction achieved from runs 1 to 6 ranged from 32% to 17% (Table 9). The best weight reduction from stage 1 is also listed and designated as run 0 for comparison.

**Figure 8:**
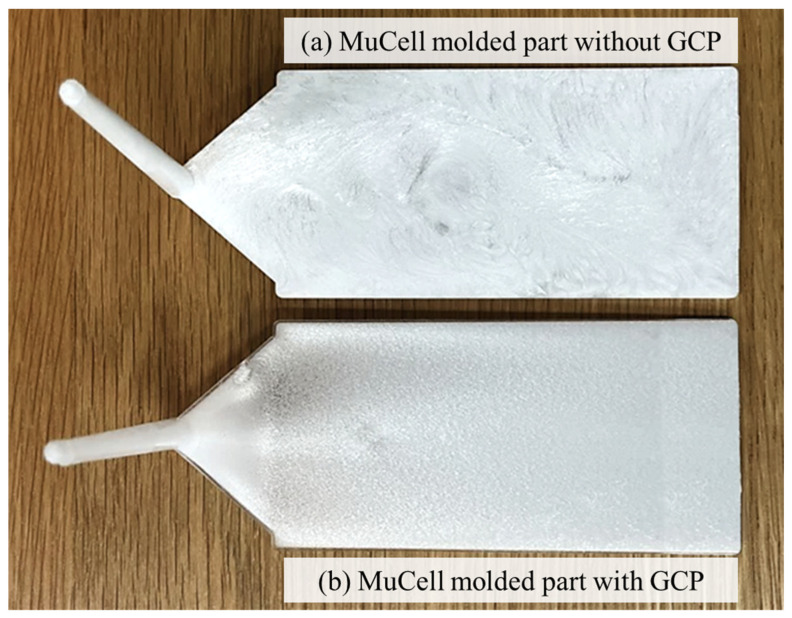
Comparison of microcellular injection-molded parts with and without GCP.

Visually, the use of GCP significantly improved the surface quality of the molded part, making it glossier and free from swirl marks and silver streaks (Figure 8).

**Figure 9:**
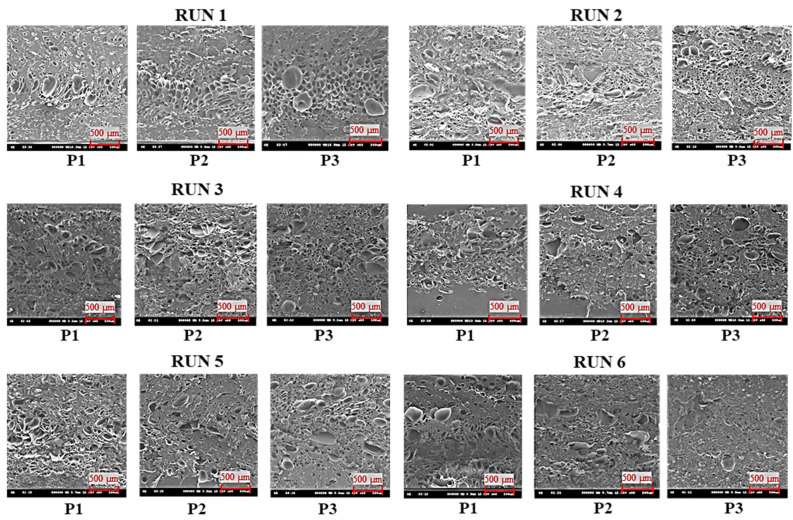
SEM images of cell structure in second stage of experiment.

**Figure 10:**
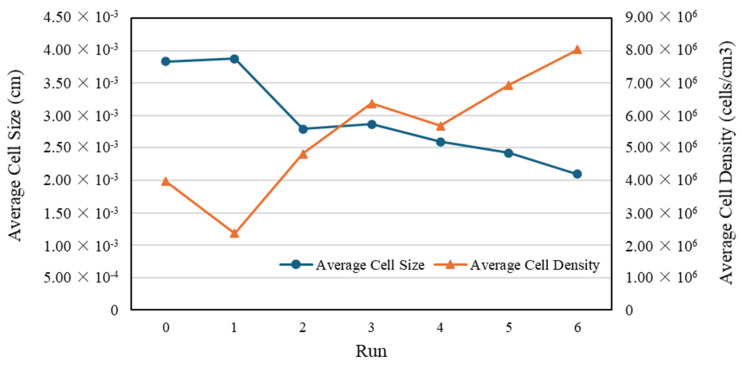
Trends in average cell size and density in second stage of experiment (run 0 represents the optimum run from the first-stage experiment).

Based on the SEM images in Figure 9, the average cell size and density calculation results for each measurement position in the second stage of the experiment are summarized in Table 10. Figure 10 shows the average cell size and density in each experimental run, including the best optimum run from the first stage (designated as run 0). Obviously, the employment of gas counter pressure and optimization of the gas holding time improve the foaming quality (i.e., decreasing the foaming cell size and increasing the cell density) significantly.

**Figure 11:**
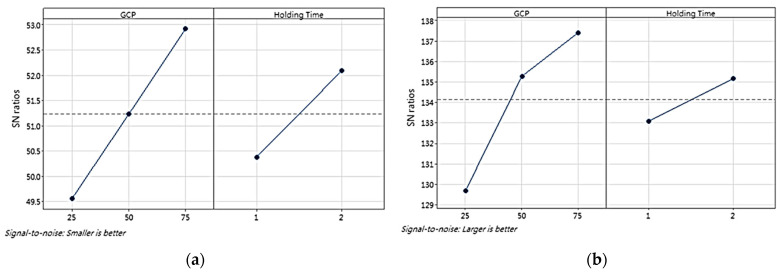
Main-effects plot of S/N ratios for (**a**) cell size and (**b**) density (P1, P2, and P3) in second stage of experiment. The dashed line represents the overall mean S/N ratio across all factor levels.

Figure 11 shows the main-effects plots of the S/N ratios for cell size and density from the Taguchi design analysis. ANOVA was conducted to evaluate the significance of each factor tested on the cell size and density. Table 11 and Table 12 provide the ANOVA results for the average cell size and density, respectively.

The Taguchi design analysis revealed a consistent pattern between the processing parameters of the GCP and the holding time in the analysis of the cell size and density; therefore, GRA was not required in the second stage. The highest S/N ratios were observed at 75 bar, indicating that increased pressure enhanced the formation of small cells, thereby increasing the overall cell density. Similarly, long holding times (2 s) contributed to high cell densities, as indicated by the high S/N ratios. This extended holding time allowed the nucleated cells to stabilize and grow uniformly, although its effect was less significant than that of the GCP. This optimal combination of GCP processing parameters corresponds to run 6.

### 3.3. Comparison of the Results from the First and Second Stages of the Experiments

Table 13 presents a comparison between the cell size and density of the molded parts with the optimal processing parameters from the first stage of the experiment (validation test/run 0 from the second stage) and those achieved after the application of GCP (run 6 from the second stage). A significant reduction in cell size was observed in the second stage, with improvements at all positions, averaging a reduction of 45.41%. Concurrently, a substantial increase in cell density was observed, with enhancements at all positions, averaging an improvement of 102.48%. Compared with the first stage of the experiment without GCP, the results showed that applying GCP resulted in smaller cells and higher cell densities.

**Figure 12:**
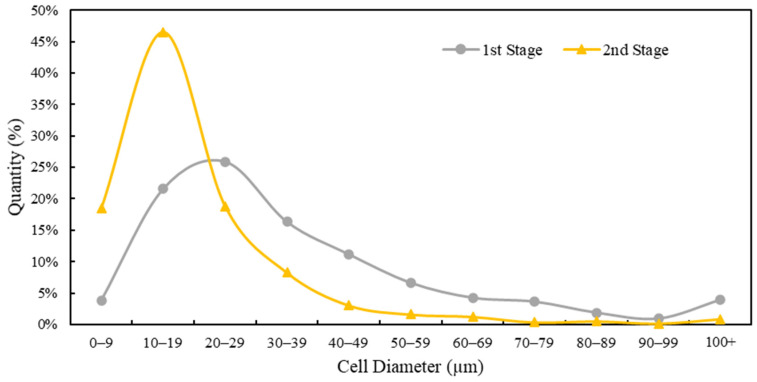
Comparison of cell size distribution between the optimum first- (validation test) and second-stage experiments (run 6).

The implementation of GCP improved the uniformity of the cells of the molded part according to a narrower cell size distribution. Figure 12 shows an overall comparison of the cell size distribution from the optimum results of the first and second stages of the experiments. The proportion of cells smaller than 20 µm drastically increased from 26% in the first stage to 64% in the second stage, indicating a 146% improvement. Additionally, 91% of the cells in the second stage were smaller than 40 µm, compared with only 68% in the first stage. This shift toward smaller cell sizes, with fewer larger cells (e.g., cells larger than 100 µm decreased from 4% to 1%), suggested that the microcellular structure was effectively refined by the GCP. The improved uniformity and reduced cell size distribution complemented the contributions of the GCP to the improved surface quality and overall stability of the material. This highlights the importance of the GCP in achieving higher quality and consistency in molded parts.

In general, the present results show that SCF injection foaming of PC materials using SCF CO_2_ can achieve a decent performance, particularly as PC is considered a very common and important engineering plastic. Compared with previous studies using PP, PS, PE, TPU (Table 1), etc., daily-necessity plastics, both the weight reduction and foaming cell are similar or even better. Products using engineering plastics require higher impact strengths; therefore, this intimately relates to the foaming cell size and their distribution. For almost 20 years since the commercialization of the MuCell^®^ process, the biggest obstacle that has hindered its application is foaming control. The current study shows thrilling results on foaming quality control in terms of the foaming cell size and their distribution and cell density. Not only this but the weight reduction also reaches about the 30~35% level at the same time. GCP has also proven to be very useful in further downsizing the foaming cell size, although this may be a compromise, with less of a weight reduction. For injection foaming of PC materials using SCF N_2_ as the blowing agent [23], although the weight reduction was able to reach 40%, its foaming quality remained to be clarified. For further improvements in the foaming quality, GCP may be combined with core-back, as proposed by Reglero et al. [31].

## 4. Conclusions

For a long time, foaming control has been a tough issue in microcellular injection molding, particularly for engineering plastic products which require a sound mechanical strength. This study successfully used SCF CO_2_ as the blowing agent in the MuCell^®^ process for PC material and produced microcellular foaming cells with sizes as low as 40 µm and cell densities reaching 3.97 × 10⁶ cells/cm^3^, with a targeted weight reduction of 35%. Gas counter pressure was further employed to downsize the cell dimensions and improve their uniformity. A high SCF dosage was found to be a significant factor in creating small cells with high cell densities, followed by reductions in the mold and melt temperatures. The injection speed had the least impact on cell size and density. Significant improvements were observed in the microcellular injection-molded PC parts using the GCP, achieving a cell size reduction of 45.41% and an increase in cell density of 102.48% compared with the parts molded without applying GCP. A high GCP value affected the cell size and density significantly, whereas an increase in the gas holding time only contributed slightly to the cell size reduction and the increase in cell density of the test parts. Additionally, GCP enhanced the uniformity of the cell size distribution, with 92% of the cells being under 40 µm and an improvement of 156% in the cells under 20 µm compared with the parts molded without GCP. For the first time in engineering PC materials, the MuCell^®^ process achieved a high foaming quality performance alongside a good weight reduction. These results will help the plastic industry to accelerate the application of MuCell^®^.

## Figures and Tables

**Table 1:** Previous studies on MuCell^®^ with supercritical fluid CO_2_ and PC material.

Research	Blowing Agent	Material	Advanced Features	Processing Parameters	Cell Size (µm)	Weight Reduction
Kaewmesri et al. [22]	CO_2_	PP		foaming temperature	3–8	
Chen et al. [23]	N_2_	PC		melt and mold temperature, microcellular plasticizing pressure, injection rate, SCF level, shot size, part thickness	Basic cell size	40%
Ishikawa et al. [24]	N_2_, CO_2_	PP	core-back	core-back rate, gas type, gas injection time, gas flow rate	100–400	
Mi et al. [25]	CO_2_	TPU		melt temperature, SCF content, injection volume and speed	190	30%
Sun et al. [13]	N_2_, CO_2_	LDPE, HDPS, PP	SCF-laden pellet	gas type, gas content	30–120	15–18%
Xi et al. [26]	CO_2_	iPP and iPP/nano-CaCO_3_		injection speed, mold temperature, gas content	25–50	15%
Wang et al. [16]	N_2_, CO_2_	PP	RIC-FIM II	delivery pressure, void fraction, gas type	N_2_: 15–30 CO_2_: 15–20	
Yeh et al. [17]	CO_2_	TPU		foaming temperature, clay content	0.45	
Wang et al. [27]	N_2_, CO_2_, Ar, He	PP	New FIM, low pressure, core-back	dwelling time, gas blowing agent, core-back distance	N_2_: 28 CO_2_: 14 Ar: 18 He: 32	
Zhao et al. [28]	CO_2_	PP/Talc	in situ fibrillation, toughening method	-	82	15%

**Table 2:** Processing parameters in first stage of experiment (without GCP).

Run	SCF Dosage (wt%)	Melt Temperature (°C)	Mold Temperature (°C)	Injection Speed (mm/s)
1	1	290	80	50
2	1	300	90	75
3	1	310	100	100
4	1.5	290	90	100
5	1.5	300	100	50
6	1.5	310	80	75
7	2	290	100	75
8	2	300	80	100
9	2	310	90	50

**Table 3:** Summary of cell size and density in first stage of experiment.

Run	Cell Size				Cell Density			
	P1	P2	P3	Average	P1	P2	P3	Average
1	5.882	5.565	4.332	5.259	0.910	1.453	2.400	1.588
2	5.043	7.379	5.663	6.028	0.487	0.862	1.756	1.035
3	7.061	8.693	6.337	7.363	0.804	0.671	0.792	0.756
4	5.264	4.125	5.128	4.839	0.938	3.159	1.971	2.022
5	8.204	5.487	4.121	5.937	0.379	1.123	2.715	1.405
6	4.410	4.556	5.591	4.852	2.673	1.992	1.563	2.076
7	4.641	4.099	4.880	4.540	2.366	2.723	1.518	2.202
8	5.998	3.910	4.163	4.691	1.190	1.762	3.267	2.073
9	5.163	3.799	5.744	4.902	1.785	3.075	1.430	2.096

Cell size (×10^−3^ cm); cell density (×10^6^ cells/cm^3^).

**Table 4:** Analysis of variance in average cell size in first stage of experiment.

Source	Degrees of Freedom (DFs)	Contribution	Adjusted Sums of Squares (SS)	Adjusted Mean Squares (MS)	F-Value	*p*-Value
Regression	4	91.16%	0.000006	0.000002	10.31	0.022
SCF Dosage	1	51.10%	0.000003	0.000003	23.11	0.009
Melt Temperature	1	15.38%	0.000001	0.000001	6.96	0.058
Mold Temperature	1	23.10%	0.000002	0.000002	10.45	0.032
Injection Speed	1	1.58%	0.000000	0.000000	0.71	0.446
Error	4	8.84%	0.000001	0.000000		
Total	8	100.00%				

**Table 5:** Analysis of variance in average cell density in first stage of experiment.

Source	DFs	Contribution	Adj. SS	Adj. MS	F-Value	*p*-Value
Regression	4	87.46%	1.95 × 10^12^	4.87 × 10^11^	6.97	0.043
SCF Dosage	1	67.06%	1.49 × 10^12^	1.49 × 10^12^	21.39	0.010
Melt Temperature	1	5.85%	1.30 × 10^11^	1.30 × 10^11^	1.87	0.244
Mold Temperature	1	14.12%	3.14 × 10^11^	3.14 × 10^11^	4.50	0.101
Injection Speed	1	0.43%	9.50 × 10^9^	9.50 × 10^9^	0.14	0.731
Error	4	12.54%	2.79 × 10^11^	6.98 × 10^10^		
Total	8	100.00%				

**Table 6:** Gray relational grades and ranks in first stage of experiment.

Run	GRG	Rank
1	0.587	6
2	0.459	8
3	0.363	9
4	0.672	5
5	0.570	7
6	0.704	3
7	0.745	2
8	0.752	1
9	0.699	4

**Table 7:** Comparison of cell size and density from run 8 with validation test.

Item	Position	Run 8	Validation Test	% Improvement
Cell size (cm)	P1	5.998 × 10^−3^	3.847 × 10^−3^	35.87%
	P2	3.910 × 10^−3^	3.813 × 10^−3^	2.50%
	P3	4.163 × 10^−3^	3.848 × 10^−3^	7.58%
	Average	4.691 × 10^−3^	3.836 × 10^−3^	18.23%
Cell density (cells/cm^3^)	P1	1.190 × 10^6^	2.661 × 10^6^	123.65%
	P2	1.762 × 10^6^	3.791 × 10^6^	115.09%
	P3	3.267 × 10^6^	5.464 × 10^6^	67.23%
	Average	2.073 × 10^6^	3.972 × 10^6^	91.59%

**Table 8:** Processing parameters in second stage of experiment (with GCP).

Run	GCP (Bar)	Holding Time (s)
1	25	1
2	25	2
3	50	1
4	50	2
5	75	1
6	75	2

**Table 9:** Weight reduction in second stage of experiment (with GCP).

Run	GCP (Bar)	Holding Time (s)	Actual Weight Reduction
0	0	0	35%
1	25	1	32%
2	25	2	28%
3	50	1	28%
4	50	2	22%
5	75	1	21%
6	75	2	17%

**Table 10:** Summary of cell size and density in second stage of experiment.

Run	Cell Size				Cell Density			
	P1	P2	P3	Average	P1	P2	P3	Average
1	2.992	3.648	4.984	3.875	3.157	2.363	1.583	2.368
2	2.815	2.958	2.607	2.793	3.785	4.097	6.574	4.819
3	2.514	2.989	3.099	2.867	6.566	6.210	6.361	6.379
4	3.099	2.339	2.340	2.592	5.865	4.154	7.028	5.682
5	2.301	2.205	2.760	2.422	7.425	7.236	6.151	6.937
6	2.055	2.221	2.006	2.094	8.204	7.572	8.351	8.042

Cell size (×10^−3^ cm); cell density (×10^6^ cells/cm^3^).

**Table 11:** Analysis of variance in average cell size in second stage of experiment.

Source	DF	Contribution	Adj. SS	Adj. MS	F-Value	*p*-Value
Regression	2	88.63%	0.000002	0.000001	11.69	0.038
GCP	1	62.93%	0.000001	0.000001	16.60	0.027
Holding Time	1	25.69%	0.000000	0.000000	6.78	0.080
Error	3	11.37%	0.000000	0.000000		
Total	5	100.00%				

**Table 12:** Analysis of variance in average cell density in second stage of experiment.

Source	DF	Contribution	Adj. SS	Adj. MS	F-Value	*p*-Value
Regression	2	85.47%	1.65 × 10^13^	8.27 × 10^12^	8.82	0.055
GCP	1	78.43%	1.52 × 10^13^	1.52 × 10^13^	16.19	0.028
Holding Time	1	7.04%	1.36 × 10^12^	1.36 × 10^12^	1.45	0.314
Error	3	14.53%	2.81 × 10^12^	9.38 × 10^11^		
Total	5	100.00%				

**Table 13:** Comparison of cell size and density from the optimum results of the first and second stages of the experiment.

Item	Position	Optimum First Stage	Optimum Second Stage	% Improvement
Cell size (cm)	P1	3.847 × 10^−3^	2.301 × 10^−3^	40.19%
	P2	3.813 × 10^−3^	2.221 × 10^−3^	41.74%
	P3	3.848 × 10^−3^	2.006 × 10^−3^	47.87%
	Average	3.836 × 10^−3^	2.094 × 10^−3^	45.41%
Cell density (cells/cm^3^)	P1	2.661 × 10^6^	7.425 × 10^6^	179.02%
	P2	3.791 × 10^6^	7.572 × 10^6^	99.74%
	P3	5.464 × 10^6^	8.351 × 10^6^	52.84%
	Average	3.972 × 10^6^	8.042 × 10^6^	102.48%

## Data Availability

Data are contained within the article.
